# Altered pain perception in children with chronic tension-type headache

**DOI:** 10.1186/1129-2377-14-S1-P17

**Published:** 2013-02-21

**Authors:** AL Soe, LL Thomsen, B Tornoe, L Skov

**Affiliations:** 1 Children´s Headache Clinic, Department of Paediatrics, Herlev University Hospital, Copenhagen, Denmark; 2 Department of Neuropaediatrics, Rigshospitalet, Copenhagen University Hospital, Copenhagen, Denmark; 3 Department of Paediatrics, Herlev University Hospital, Copenhagen, Denmark

## Introduction

Children with tension-type headache (TTH) might have an altered pain perception. Some of these children suffer from the chronic form of TTH. It is not yet known if central sensitization plays a role in chronification of TTH in children.

## Objectives

The aim of this study was to use stimulus-response functions for pressure versus pain to test the difference in pain perception between children 7-17 years of age with frequent episodic tension-type headache (FETTH), chronic tension-type headache (CTTH) and controls.

## Method

From May 2009-May 2011 we included 22 children with FETTH, 36 children with CTTH and 57 controls into this case-control study. We applied pressure of 5 increasing intensities to M. Trapezius and M. Temporalis respectively with a Somedic Algometer II. The child rated pain on a VAS-scale.

## Statistical methods

Area under the curve (AUC) was calculated and represents the tenderness of the muscle. Whereas factor analysis showed that AUC represents only one dimension common for both muscles, an average AUC in each person was used as outcome variable in further univariate multiple linear regression analysis.

## Results

Stimulus-respons functions were different between the control group and CTTH. CTTH had a significant higher AUC (median 338, inter-quartile range (IQR) 180-406) than the control group (median 191, IQR 83-286) P<0.001. However AUC in the FETTH group (median 281, IQR 202-371) was not significantly different from either the control group (P=0.084) or CTTH (P=0.283), indicating that this group must represent an intermediate state between the two extreme groups. Sensitivity (AUC) did not change with increasing age, headache years, headache intensity, headache frequency or sex.

## Conclusion

Pain perception for pressure versus pain in children with CTTH is altered. These changes seem to be a continuum of changes with the FETTH representing an intermediate state between controls and children with CTTH.

